# Measuring plant cysteine oxidase interactions with substrates using intrinsic tryptophan fluorescence

**DOI:** 10.1038/s41598-024-83508-y

**Published:** 2024-12-30

**Authors:** Dona M. Gunawardana, Daisy A. Southern, Emily Flashman

**Affiliations:** 1 Department of Chemistry, University of Oxford, Oxford, OX1 3TA UK; 2 Department of Biology, University of Oxford, Oxford, OX1 3RB UK

**Keywords:** Plant sciences, Proteins, Synthetic biology, Chemical biology, Enzymes

## Abstract

Plant Cysteine Oxidases (PCOs) are oxygen-sensing enyzmes that catalyse oxidation of cysteinyl residues at the N-termini of target proteins, triggering their degradation via the N-degron pathway. PCO oxygen sensitivity means that in low oxygen conditions (hypoxia), their activity reduces and target proteins are stabilised. PCO substrates include Group VII Ethylene Response Factors (ERFVIIs) involved in adaptive responses to the acute hypoxia experienced upon plant submergence, as well as Little Zipper 2 (ZPR2) and Vernalisation 2 (VRN2) which are involved in developmental processes in hypoxic niches. The PCOs are potential targets for improving submergence tolerance through enzyme engineering or chemical treatment. To achieve this, a detailed understanding of their biological function is required. Here, we report development of an assay that exploits the intrinsic fluorescence of *Arabidopsis thaliana* PCO tryptophan residues. By using Ni(II)-substitued enzymes and preparing the assay under anaerobic conditions, tryptophan fluorescence quenching is observed on enzyme:substrate complex formation, allowing quantification of binding affinities. Our assay revealed that, broadly, AtPCO4 and AtPCO5 have stronger interactions with ERFVII substrates than ZPR2 and VRN2, suggesting ERFVIIs are primary targets of these enzymes. It also revealed a positive cooperative binding effect for interactions between AtPCOs4/5 and ERFVIIs and ZPR2. The assay is experimentally straightforward and can be used to further interogate PCO interactions with substrates.

**Supplementary Information:**

The online version contains supplementary material available at 10.1038/s41598-024-83508-y.

## Introduction

Plant Cysteine Oxidases (PCOs) are oxygen-sensing enzymes that catalyse the first step of the Cys/Arg branch of the N-degron pathway in plants. Under normoxic conditions, PCOs recognise and oxidise the methionine-cleaved N-terminal cysteine of target proteins, rendering them substrates for arginyl transferase (ATE)1/2-catalysed arginylation, proteolysis 6 (PRT6)-mediated ubiquitination and degradation by the proteasome. However, on reduction of oxygen availability (hypoxia) PCO activity decreases and target proteins are stabilised^1–3^. Group VII Ethylene Response transcription factors (ERFVIIs) are known targets of the PCOs and susceptible to oxygen-dependent destabilisation by the N-degron pathway^4–6^. In hypoxic conditions including waterlogging and submergence, stabilised ERFVIIs upregulate genes which enable hypoxic adaptation, including those encoding pyruvate decarboxylase, alcohol dehydrogenase and sucrose synthase, which promote anaerobic metabolism to maintain basal ATP production^7,8^. Additional reported PCO targets include Little Zipper 2 (ZPR2) and Polycomb group protein Vernalisation 2 (VRN2); stabilisation of these targets in plant hypoxic niches enables developmental control (VRN2 enables methylation-induced silencing of *FLOWERING LOCUS C* while ZPR2 regulates the activity of transcription factors critical for shoot apical meristem activity)^9,10^. Overall, PCOs connect the availability of O_2_ with critical responses to hypoxia^2,3,11^. They are important targets for modulation via engineering or inhibition to positively impact plant submergence tolerance, particularly as crop yields are increasingly challenged by climate change^7,12,13^.

Structures have been experimentally solved for *Arabidopsis thaliana* PCOs 2, 4 and 5 which reveal double-stranded beta helix scaffolds supporting a 3 × His triad that coordinates an active site metal (Fe(II))^14,15^. No substrate-bound structures have been solved to date, however spectroscopic and in silico studies in a mammalian paralogue of the PCOs, 2-aminethanethiol dioxygenase (ADO)^16^, raises the possibility that the thiol of Nt-Cys initiating substrates coordinates to the active site metal, while the Nt-amino group interacts with a nearby Asp residue^17–19^. Modelled AtPCO interactions with a short peptide representing the Cys-initiating N-terminus of the Arabidopsis ERFVII RAP2.12 indicate these interactions are also important in PCOs^20^. A conserved hairpin loop (residues 182–190 in AtPCO4) may also be involved with substrate binding and selectivity^15^. Although all PCO substrates contain N-terminal Cys residues, the residues following the Nt-Cys are not necessarily conserved; ERF-VIIs (of which there are 5 in *Arabidopsis thaliana*; RELATED TO AP-2 (RAP2)0.12, RAP2.2, RAP2.3, HRE1 and HRE2) share conserved N-terminal sequences of CGGA(I/V)ISD(F/Y) but VRN2 and ZPR2 are entirely different (Supplementary Table 1)^9,10,21,22^. Furthermore, there are over 200 additional Met-Cys initiating proteins in the Arabidopsis proteome which all have the potential to be PCO targets (albeit structurally inaccessible N-termini or other N-terminal modifications may prevent this)^4,23^. For PCO modulation strategies to be effective it is necessary to understand how these enzymes interact with their substrates. While kinetic assays are well established for the PCOs^11^, a substrate-binding assay is not. Such an assay has the potential to reveal both biological insight into preferred and novel PCO substrates but also the impact of inhibitors or mutations on these interactions.

Fluorescence assays are widely used for protein–ligand binding quantification. In the absence of fluorescent ligands (which can be expensive and/or compromise binding characteristics), protein intrinsic fluorescence can be exploited, arising from aromatic residues, predominantly tryptophan^24,25^. Tryptophan residues emit fluorescence at 300–400 nm when excited at 280 nm in a manner that is sensitive to their micro-environment, meaning fluorescence intensity can change upon interaction with ligands^25^*.* Therefore, intrinsic tryptophan fluorescence (ITF) quenching can be an excellent tool to study protein–ligand interactions as well as to determine enzyme–substrate binding affinities. Reported examples include 2,4-dichlorophenoxyacetic acid dioxygenase (TfdA), 1-aminocyclopropane-1-carboxylic acid oxidase (ACCO), the peptidoglycan glycosyltransferase MtgA and galactoside-binding mammalian proteins ^26–29^. Here we describe development and implementation of an ITF quenching assay to quantify PCO interaction with peptides representing known substrates ERFVIIs, VRN2 and ZPR2.

## Materials and methods

### AtPCO4/5 preparation

Expression and His_6_-tag affinity purification of AtPCO4 and 5 were as previously described^11^*.* Following affinity purification, the His_6_-tag was cleaved using TEV protease and the cleaved tag was removed using a HisTrap HP column (GE Healthcare). To remove the active site metal which co-purifies with PCO enzymes^3^, concentrated protein fractions were dialysed into 50 mM Tris (pH 7.5), 0.4 M NaCl, 100 mM EDTA and 10 mM 1,10-Phenanthroline followed by dialysis into 50 mM Tris (pH 7.5), 0.4 M NaCl and 0.1% (w/v) Chelex® 100 resin to remove excess EDTA and 1,10-Phenanthroline. Dialysed protein was then purified with a HiLoad 26/600 Superdex 75 prep grade size exclusion column (GE Healthcare) equilibrated with 50 mM Tris (pH 7.5) and 0.4 M NaCl (herein termed assay buffer). Protein purity was assessed with SDS-PAGE. To check that Fe(II) was removed, activity assays of metal-removed protein were conducted as described previously^30^.

### Sample preparation for fluorescence measurements

Metal-removed AtPCO4/5 was incubated with Ni(II) (NiCl_2_.6H_2_O, Alfa Aesar) at enzyme:metal, 1:2 ratio and diluted to the desired volume in assay buffer to a final enzyme concentration of 8 µM, termed the enzyme master mix. 14-mer peptides representing substrates AtRAP2.12_2–15_, AtHRE1_2-15_, AtVRN2_2-15_, AtZPR2_2-15_ and the peptide (GAGK)_2_PAPK(GAGK)_2_ (GL Biochem, China) were dissolved in assay buffer plus 2 mM TCEP to make appropriate stock solutions. All samples were prepared at room temperature.

**Fig. 1:**
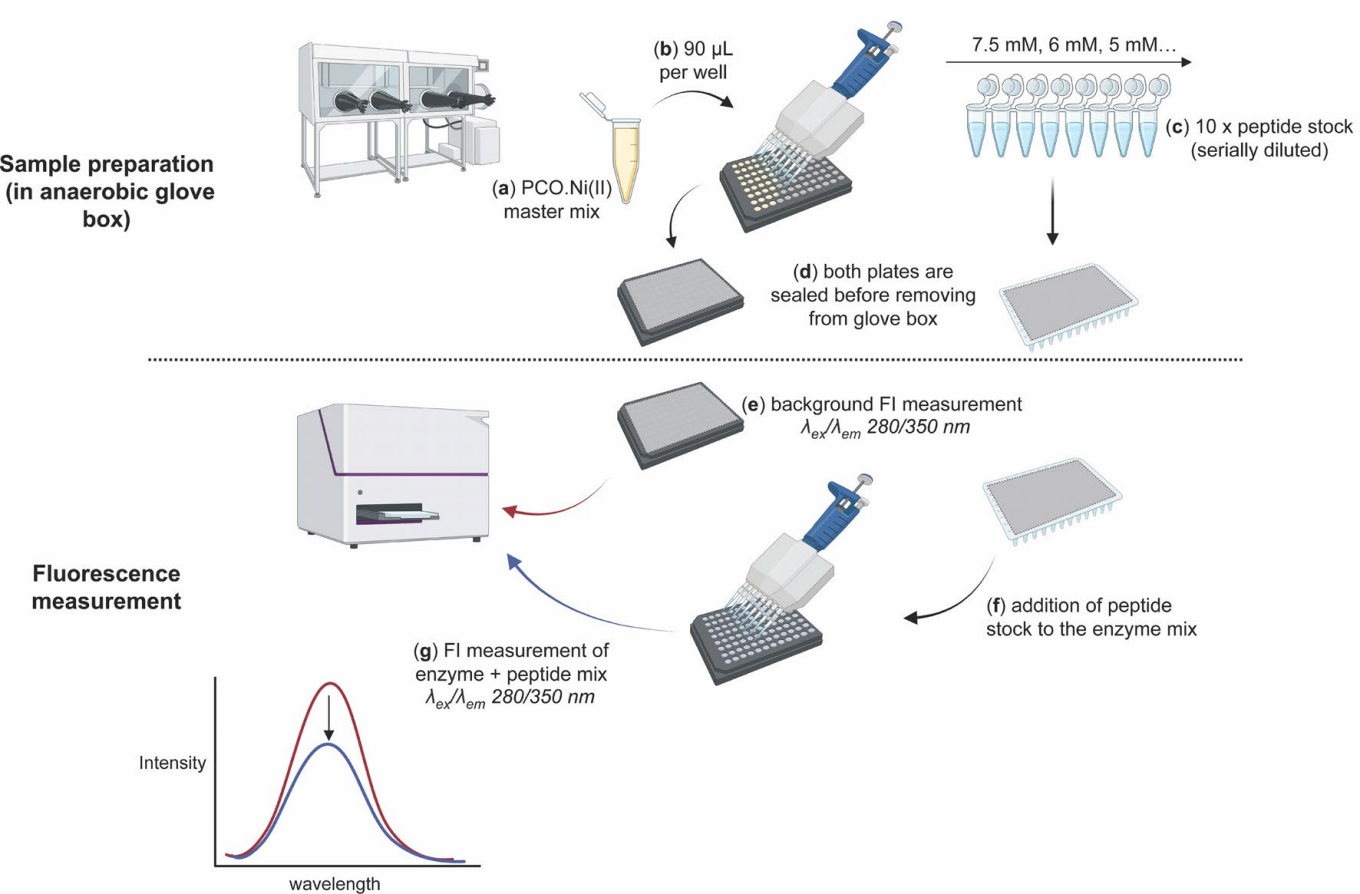
Simplified graphic illustration of the fluorescence quenching method. For sample preparation (in an anaerobic glove box at 25 °C), enzyme master mix (**a**) is prepared by mixing the enzyme with the Ni(II) at 1:2 ratio in assay buffer (50 mM Tris (pH 7.5), 0.4 M NaCl) to a final enzyme concentration of 8 µM. 90 µL of the enzyme mix is then added to each well of a black-bottom 96-well plate (assay plate, **b**). Peptide stock is serially diluted in assay buffer plus 2 mM TCEP to give 10 × the final required peptide concentration (**c**), sufficient volume of each peptide stock is transferred into a separate 96-well plate. Both plates are sealed with an aluminium foil seal (**d**). In the fluorescence measurement stage, foil seal is removed from the assay plate and background fluorescence is measured using a plate-reader, at 25 °C (**e**). 10 µL of each serially diluted peptide stock is added simultaneously to the appropriate wells of the assay plate and mixed thoroughly by pipetting (**f**). Enzyme and substrate are incubated for 10 min at 25 °C before fluorescence intensity is measured again (**g**).

For anaerobic fluorescence measurements, sample preparation took place under a nitrogen atmosphere in an anaerobic glove box (Belle Technology) (Fig. 1). Assay buffer was purged with 100% N_2_ and equilibrated overnight in the glove box prior to any sample preparation. To minimise the O_2_ in the final samples, Ni(II) and peptide stocks were made in the anaerobic glove box using the N_2_-purged assay buffer. Concentrated stocks of TCEP and enzyme were exposed to the N_2_ atmosphere of the glove box for 10 min prior to the sample preparation.

### Cuvette-based fluorescence measurements

Fluorescence emission spectra of AtPCO4.Ni(II) were measured on a Shimadzu RF-6000 spectrofluorophotometer at λ_ex_/λ_em_ 280/350 nm using a 2 mL quartz cuvette. 3 µL aliquots of AtRAP2. 12_2–15_ (at 46.87 mM) were titrated into 1.5 mL AtPCO4.Ni(II) solution (at 8 µM) and mixed thoroughly by pipetting. To investigate the impact of oxygen on fluorescence emission, 1.5 mL AtPCO4.Ni(II) (at 8 µM) was prepared in an anaerobic glove box, transferred to a 2 mL quartz cuvette and sealed with a gas tight cap. Fluorescence emission spectrum of the anaerobic AtPCO4.Ni(II) solution was measured on a Shimadzu RF-6000 spectrofluorophotometer at λ_ex_/λ_em_ 280/350 nm. Compressed air was then bubbled through the cuvette for 2 min. Following the treatment, fluorescence emission spectrum was recorded after 0, 1 and 2 min.

### Plate-based fluorescence measurements

AtPCO4/5-Ni(II) enzyme master mix was prepared in assay buffer (enzyme:metal, 1:2 ratio); for each sample, 90 µL of the master mix was transferred to one well of a 96-well black flat-bottom microplate (Greiner bio-one), termed ‘assay plate’ (Fig. 1a,b). Peptide substrate solutions were prepared in assay buffer plus 2 mM TCEP, at a range of concentrations decreasing from 7.5 to 0 mM (Fig. 1c,d). These were transferred to a separate plate. If being prepared in an anaerobic glove box, both plates were sealed before removal.

Fluorescence intensity was measured at λ_ex_/λ_em_ 280/350 nm using an optic filter module (FI 280 350, 1304A1) in a BMG PHERAstar microplate reader. Instrumental parameters were kept consistent throughout all measurements (dynamic adjustment range (530–600), gain adjustment (531) and temperature (25 °C)). For each experiment, plate seals were removed and background fluorescence intensity of the assay plate (AtPCO4/5.Ni(II) only) was measured (Fig. 1e). Equal volumes (10 µL) of peptide stock solution were then added to each well containing the enzyme mix, giving final peptide concentrations ranging from 750 to 0 µM (Fig. 1f). Samples were incubated for 10 min at 25 °C prior to a second fluorescence intensity measurement (Fig. 1g). For inner filter effect analysis, sample preparation and fluorescence measurements with bovine serum albumin (BSA) (Merck) and RAP2.12_2–15_ were carried out in the same way as those with AtPCO4/5 described above.

### Data analysis

The difference between enzyme-only (background) and enzyme + substrate (sample) fluorescence measurement was determined. Sample fluorescence intensity was then corrected for 0 µM substrate and the percentage quenching was calculated. Corrected percentage quenching was plotted against the peptide concentration and fitted with either a one site- specific binding or a one site- specific binding with Hill slope plot to determine the K_D_ using GraphPad Prism (ver. 10.1.1).

## Results

### Fluorescence of two PCO Trp residues can be exploited to measure substrate binding

**Fig. 2:**
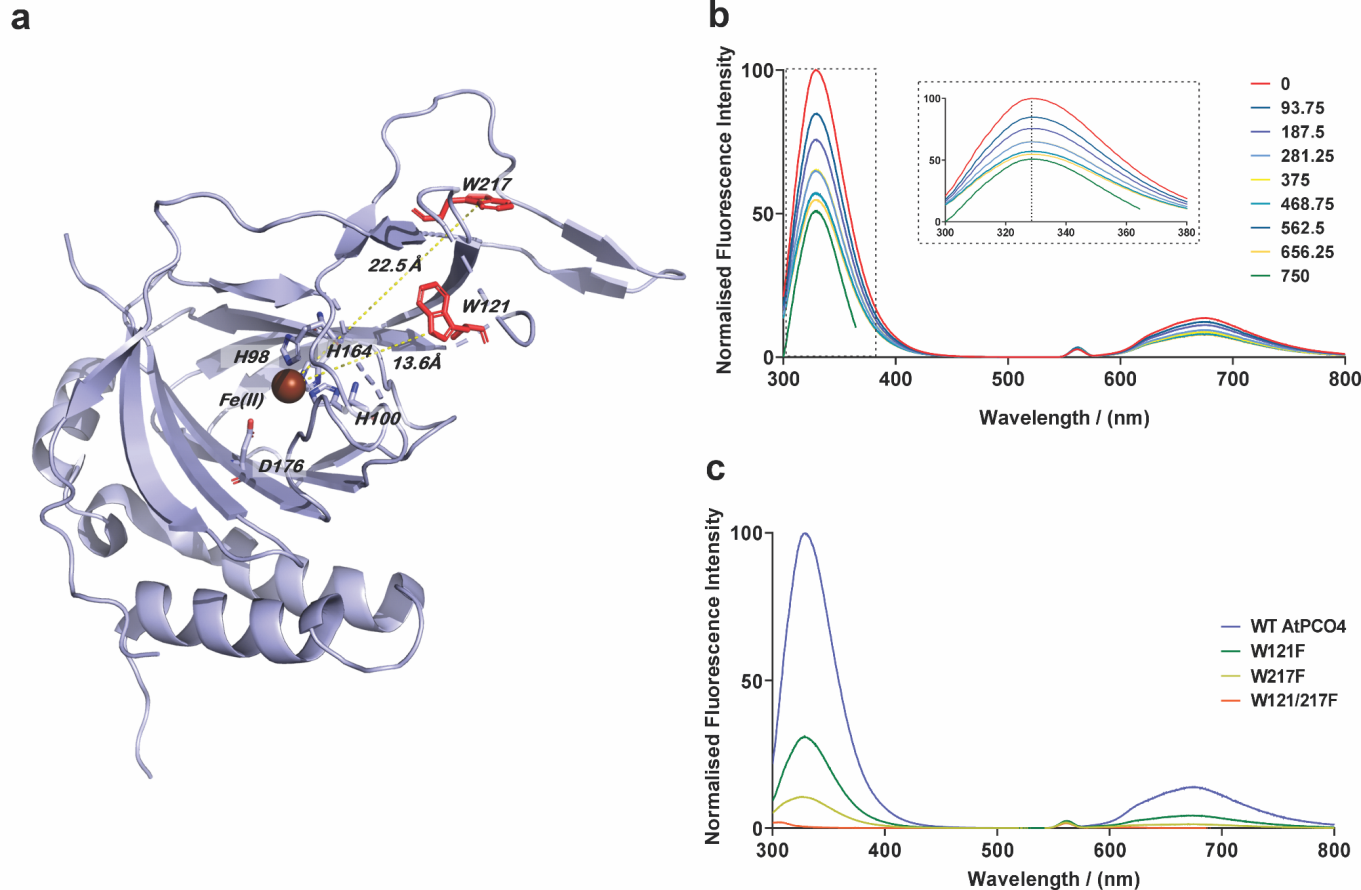
Development of a Trp-Fluorescence Quenching Assay for AtPCO4. (**a**) Structure of AtPCO4 (PDB ID: 6S0P) showing position of Trp residues 121 and 217 relative to the active site shown by the Fe(II) coordinated by the 3 × His residues. (**b**) Concentration-dependent Trp fluorescence quenching was observed upon titration of RAP2_2-15_ solution (final concentrations ranging from 93.5 to 750 µM) into 8 µM AtPCO4.Ni(II). (**c**) Fluorescence intensity decreases when the two Trp residues, 121 and 217 are mutated to Phe confirming their contribution to observed fluorescence intensity.

The 5 PCO enzymes from Arabidopsis all have at least 2 tryptophan residues, with Trp residues W121 and W217 being conserved (AtPCO4 numbering) (Supplementary Fig. 1). Analysis of the AtPCO4 and AtPCO5 structures^14,15^ as well as the modelled structure of AtPCO4 in complex with a peptidic form of the RAP2.12 substrate^20^, indicates that these Trp residues are in a region of the enzyme that comprises a significant proportion in an unstructured/flexible conformation, outside of the canonical double-stranded beta helix core fold typical of the thiol dioxygenases and other cupin superfamily proteins^31^ (Fig. 2a). We considered it possible that these regions may undergo a conformational change upon substrate binding and therefore that the AtPCO Trp fluorescence properties might be different with or without substrate. This could allow Trp fluorescence quenching to be used as a measure of the strength of substrate binding.

We sought to test this using recombinant AtPCO4 and a 14-mer peptide, RAP2_2-15_, representing AtRAP2.12 (and RAP2.2, Supplementary Table 1) as a model system. In order to generate a stable AtPCO4.RAP2_2-15_ complex, it was necessary to prepare recombinant AtPCO4 in which the active site metal, Fe(II), was removed and replaced with catalytically inactive Ni(II) to prevent any substrate turnover. This was achieved using an EDTA and 1,10-phenanthroline incubation step prior to size exclusion chromatography; the resultant enzyme was confirmed to be inactive (Supplementary Fig. S2). The Trp fluorescence of AtPCO4.Ni(II) complex (with AtPCO4 at 8 µM and Ni(II) at 16 µM) was then measured in a cuvette-based assay, using an excitation wavelength of 280 nm and monitoring the fluorescence emission spectrum. A large fluorescence emission signal was observed in the range of 300–400 nm, typical of Trp fluorescence^25^. Titration of a solution containing RAP2_2-15_ into AtPCO4.Ni(II) up to 750 µM resulted in quenching of the AtPCO4.Ni(II) fluorescence signal in a concentration-dependent manner (Fig. 2b). A control experiment in which equivalent volumes of assay buffer only were added allowed us to ascribe the Trp fluorescence quenching to RAP2_2-15_ binding to AtPCO4, rather than a dilution effect (Supplementary Fig. S3). Notably, there was no discernible shift in the maximum emission wavelength upon RAP2_2-15_ binding (Fig. 2b, insert) indicating that there were minimal changes in the hydrophobicity of the Trp local environments on substrate binding^25^.

To confirm that the two AtPCO4 Trp residues were responsible for the fluorescence changes, single and double Trp PCO variants replacing each Trp with Phe were generated; W121F, W217F and W121/217F. As anticipated, the AtPCO4 W121/217F double Trp variant showed no significant fluorescence signal (Fig. 2c). Both AtPCO4 W121F and W217F single Trp variants produced fluorescence spectra, albeit with reduced signal intensity compared to the wild type AtPCO4. W217F showed the weakest fluorescence intensity suggesting W217 contributes more significantly than W121 to the overall fluorescence observed with wild type AtPCO4 (Fig. 2c).

### Optimisation of a plate-based method for measuring Trp-fluorescence quenching upon substrate binding to AtPCO4

Having shown the potential for Trp fluorescence quenching to be used to measure PCO substrate binding, we next wanted to move from high-volume cuvette-based measurements to low-volume plate-based measurements to enable a high throughput binding assay to be established. Enzyme:substrate incubations were therefore conducted in 96-well plates with Trp fluorescence measurements taken in a plate-reader using fluorescence filters with excitation/emission (λ_ex/_λ_em_) wavelengths of 280/350 nm. Given the smaller assay volumes used in microplates (100 µL), titration of increasing volumes of substrate into individual wells would have resulted in significant dilution. A ‘separate well’ method was therefore adopted where a different well was used for stock solutions of each substrate at a range of different concentrations (Fig. 1). Background fluorescence (enzyme-only) was measured before equal volumes of substrate (at different concentrations) were simultaneously added to separate wells containing 8 µM AtPCO4.Ni(II). This method was used to conduct further controls and optimise the assay.

**Fig. 3:**
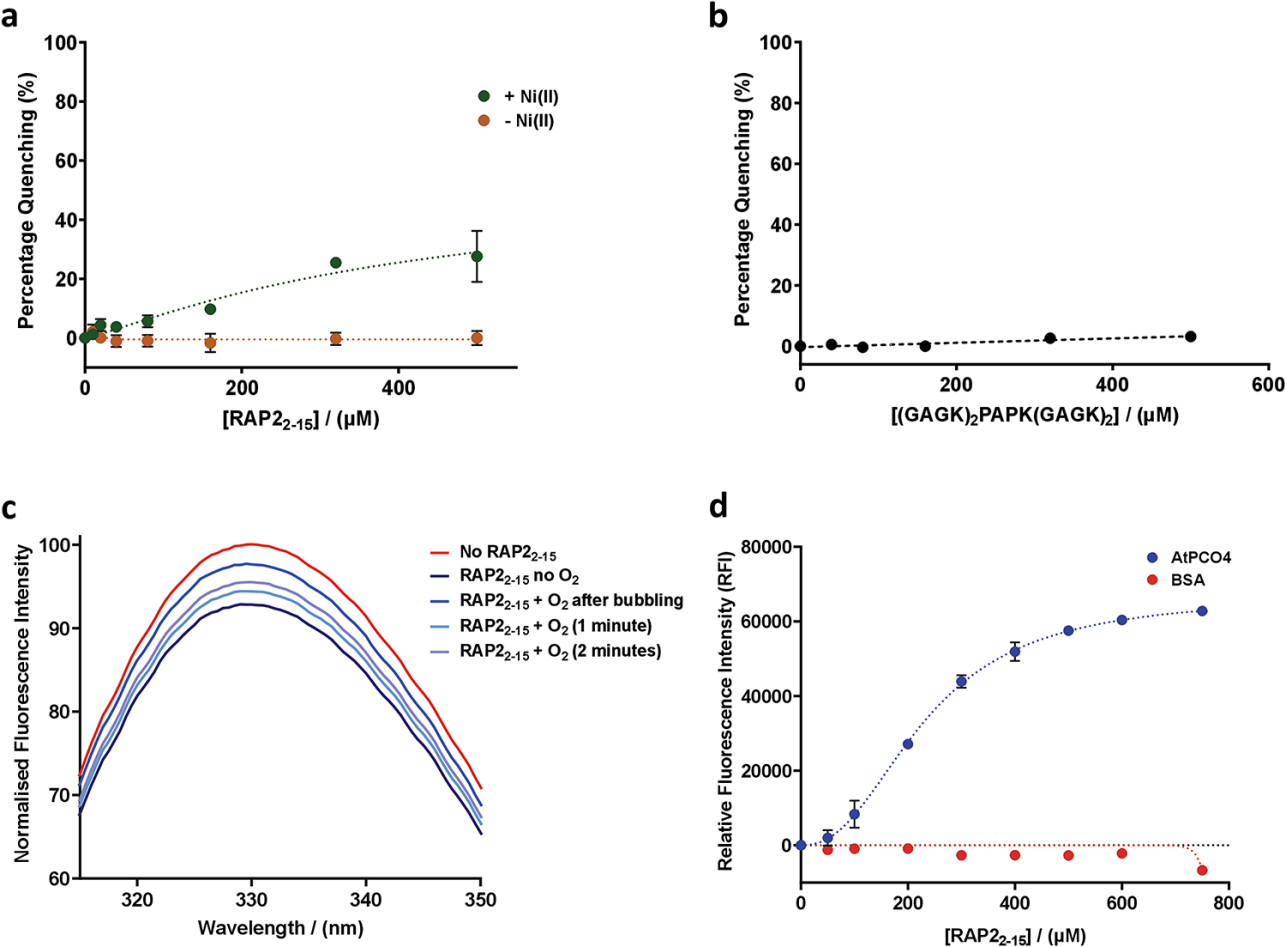
Control experiments confirm Trp-Fluorescence Quenching of AtPCO4 is substrate-dependent. (**a**) Addition of 0–500 µM RAP2_**2–15**_ to 8 µM AtPCO4 without Ni(II) supplementation showed no Trp fluorescence quenching compared to 8 µM AtPCO4 with Ni(II) at 1: 2 ratio. (**b**) Addition of a non-binding peptide, (GAGK)_**2**_PAPK(GAGK)_**2**_, to 8 µM AtPCO4.Ni(II) showed no significant concentration-dependent fluorescence quenching (**c**) Introduction of O_**2**_ (by bubbling compressed air into the sample) to a cuvette containing anaerobic AtPCO4.Ni(II) + RAP2_**2–15**_ resulted in increased fluorescence intensity. (**d**) Addition of 0–750 µM RAP2_**2–15**_ to 8 µM of either AtPCO4.Ni(II) or BSA showed negligible inner filter effects with increasing concentration of RAP2_2–15_. *Error bars display S.E *(*n* = 3)*.*

Given the distance between AtPCO4 W217 and W121 and the active site metal to which substrate is assumed to bind, we next wanted to check that Trp fluorescence quenching was due to productive RAP2_2-15_ binding. RAP2_2-15_ was therefore titrated into AtPCO4 in the presence and absence of added metal (Ni(II)). Trp fluorescence quenching increased with additional RAP2_2-15_ in the presence of Ni(II), but no quenching was observed in the absence of Ni(II) (Fig. 3a), consistent with RAP2_2-15_ binding in a productive manner, likely with its N-terminal Cys coordinating to the active site metal as previously indicated^18,19^. Fluorescence quenching was next shown to be specific to the addition of known AtPCO substrates, through the titration of a non-binding peptide to AtPCO4.Ni(II) (a viral collagen prolyl hydroxylase substrate, (GAGK)_2_PAGK(GAGK)_2_^32^). This did not result in a concentration-dependent reduction in Trp fluorescence intensity, confirming the specificity of the quenching effect (Fig. 3b).

The presence of oxygen was considered to be a potential confounding factor in the assay as it is a known quencher of Trp fluorescence^33^. In addition, as O_2_ is a co-substrate of AtPCO4, it cannot be ruled out that O_2_ binds to the AtPCO4.Ni(II).RAP2_2-15_ complex, impacting its structure and therefore Trp fluorescence properties. To test for the impact of O_2_, we prepared samples for cuvette-based fluorescence spectrum measurement in an anaerobic glove box. When RAP2_2-15_ was titrated into AtPCO4 in the absence of O_2,_ fluorescence quenching was observed as expected. Interestingly, upon exposing the sample to air (by bubbling compressed air into the sample), no further fluorescence quenching was observed, however fluorescence intensity gradually increased and fluctuated in a time-dependent manner (Fig. 3c), potentially due to conformational changes arising on O_2_-binding. For the purposes of developing a binding assay, samples for the microplate-based method were subsequently prepared anaerobically to minimise exposure to air.

Absorption of incident or emitted radiation in samples with a high concentration of fluorophores can result in a decrease in fluorescence intensity, known as the inner filter effect (IFE)^25,34,35^. This can impact the intensity of fluorescence quenching upon ligand binding. Any inner filter effect caused by addition of variable concentrations of substrate to AtPCO4.Ni(II) samples could therefore impact determination of binding constants. Absorption of radiation at 280 or 350 nm by substrate was not anticipated given that the peptides used in this study did not contain Trp or Tyr residues likely to absorb in this region^36^. Furthermore, the decreased path length in the plate-based assay (2.94 mm vs. 1 cm for conventional cuvette used for spectrophotometer) reduces the absorbance of the sample and therefore minimizes the impact of any inner filter effect^35^. Nevertheless, we still checked whether such an effect could be observed for RAP2_2-15_. For this, we replaced AtPCO4.Ni(II) with inert bovine serum albumin (BSA), which has three intrinsic Trp residues, has previously been used as a reference fluorophore for other Trp fluorescence binding assays^29^, and is not expected to interact with RAP2_2-15_. We measured fluorescence upon addition of increasing concentrations of RAP2_2-15_ to BSA and observed no significant change in relative fluorescence intensity. This indicated a negligible inner filter effect in the presence of increasing concentrations of RAP2_2-15_, particularly when compared to the degree of AtPCO4 fluorescence quenching observed under the same conditions (Fig. 3d). It was therefore deemed unnecessary to carry out inner filter effect corrections for subsequent assays although we would recommend checking for inner filter effects in assays utilising peptides containing Trp or Tyr residues^35^. Independent of inner filter effects, we included a substrate correction step (subtraction of fluorescence arising from substrate only) for all binding assays to ensure accurate binding constant determination.

### Determination of binding constants for AtPCO4 and 5 interactions with known substrates

We were then able to apply the optimised plate-based method to determination of binding constants for AtPCO enzymes with peptides representing the N-termini of their biological substrates. Trp fluorescence intensity was first measured upon addition of 0 to 750 µM peptide substrate to 8 µM AtPCO4.Ni(II) under anaerobic conditions. Fluorescence intensity levels were corrected by subtracting background (AtPCO4.Ni(II) only) and substrate-only fluorescence intensity levels. Fluorescence quenching was then calculated as a percentage of Trp fluorescence for AtPCO4.Ni(II) only, plotted against the concentration of added substrate. K_D_ values were determined by fitting specific binding models to the data.

**Fig. 4:**
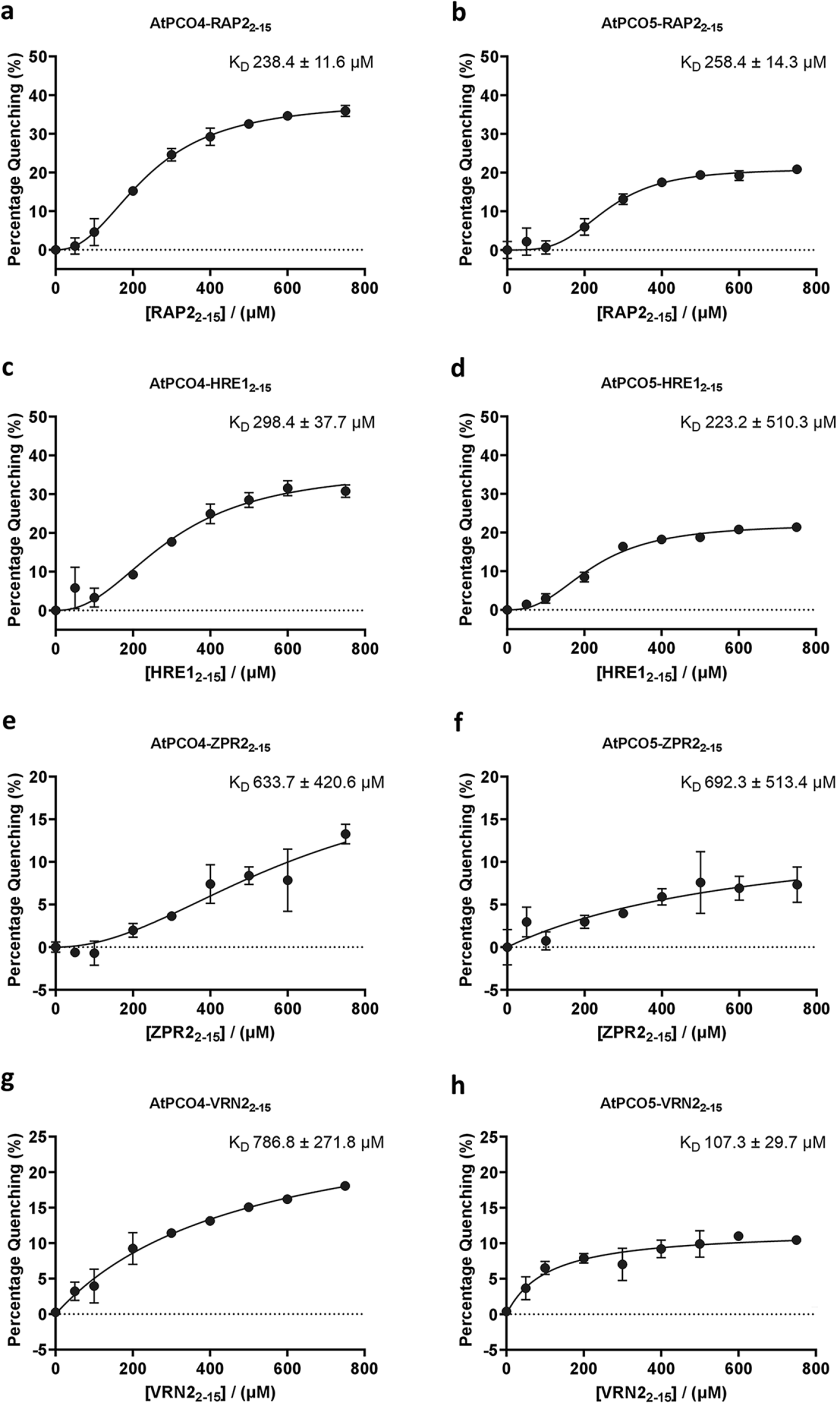
Determination of binding constants for (**a**,**c**,**e**,**g**) AtPCO4.Ni(II) and (**b**,**d**,**f**,**h**) AtPCO5.Ni(II) with peptides representing known substrates. Binding curves for AtPCO4/5 with peptides representing Cys-initiating residues 2–15 of (**a**, **b**) RAP2.12, (**c**, **d**) HRE1, (**e**, **f**) ZPR2 and (**g**, **h**) VRN2. Peptide concentrations ranging from 0 to 750 µM with 8 µM of enzyme in assay buffer. *Error bars display S.E *(*n* = 3)*.* Data are fitted to equations representing a specific binding model ((Y = Bmax*X/(K_D_ + X), **a**–**e**) or a specific binding model incorporating a Hill slop (Y = Bmax*X^h/(Kd^h + X^h), **f**–**h**).

These assays were first conducted for AtPCO4.Ni(II) with peptides RAP2_2-15_ and HRE1_2-15_, representing ERF-VIIs, as well as VRN2_2-15_ and ZPR2_2-15_ (sequences in Supplementary Table S1). Interestingly, binding curves for RAP2_2-15_, HRE1_2-15_ and ZPR2_2-15_ all fitted a specific binding model incorporating a Hill slope (Y = Bmax*X^h/(Kd^h + X^h), suggesting a cooperative binding mode for these substrates (Fig. 4a–c). Determined K_D_ values (Table 1) were 238 ± 12 µM and 298 ± 38 µM for RAP2_2-15_ and HRE1_2-15_, respectively, with ZPR2_2-15_ apparently binding more weakly (K_D_ = 634 ± 421 µM). Greater cooperativity was observed for the peptides which bound more strongly (Hill slopes of 2.3 for both RAP2_2-15_ and HRE1_2-15_) than for ZPR2_2-15_ (Hill slope of 2.0). The binding curve for VRN2_2-15_ fit a simple binding model ((Y = Bmax*X/(K_D_ + X)) with no apparent cooperativity (Fig. 4d) and also revealed weaker binding to AtPCO4.Ni(II) than RAP2_2-15_ and HRE1_2-15_, with a K_D_ of 495 ± 98 µM.

**Table 1:** Binding parameters of AtPCO4.Ni(II) and AtPCO5.Ni(II) with RAP2_2-15_, HRE1_2-15_, ZPR2_2-15_ and VRN2_2-15_.

Substrate	AtPCO4		AtPCO5	
	Kd (µM)	Curve fitting	Kd (µM)	Curve fitting
RAP2_2-15_	238.4 ± 11.6	Allosteric	258.4 ± 14.3	Allosteric
HRE1_2-15_	298.4 ± 37.7	Allosteric	223.2 ± 10.3	Allosteric
ZPR2_2-15_	633.7 ± 420.6	Allosteric	692.3 ± 513.4	Simple
VRN2_2-15_	495.0 ± 98.4	Simple	107.3 ± 29.7	Simple

Data used to generate binding parameters shown in Fig. 4

AtPCO5 has a high degree of sequence conservation with AtPCO4, including the two Trp residues W121 and W217 (Supplementary Fig. S1). While AtPCO5 has an additional Trp residue close to the N-terminal alpha-helical region (AtPCO5 W54), we nevertheless considered it likely that the Trp fluorescence quenching assay would have the same capability to measure substrate binding as it does for AtPCO4. Interestingly, one region of difference between AtPCO4 and AtPCO5 is in a hairpin loop (residues 182–190 for both AtPCO4 and AtPCO5) which comprises charged and polar residues^15^. This loop has previously been proposed to be involved in substrate binding and possesses two negatively charged residues for AtPCO4 (E185 and D187); these residues are neutral in AtPCO5 (T185 and G187)^15^. This may impact the interactions of these enzymes with different substrates^11^. To investigate this, we therefore conducted binding assays with AtPCO5.Ni(II) under equivalent conditions to those used for AtPCO4.

Binding of RAP2_2-15_ and HRE1_2-15_ to AtPCO5.Ni(II) revealed very similar binding constants to those determined for AtPCO4 (258 ± 14 µM and 223 ± 10 µM for RAP2_2-15_ and HRE1_2-15_, respectively, Fig. 4b,d and Table 1), albeit with AtPCO5.Ni(II) appearing to show a slightly greater affinity for HRE1_2-15_ over RAP2_2-15_, in contrast to AtPCO4.Ni(II) but consistent with substrate preferences previously observed in enzyme activity assays^11^. These interactions again showed a cooperative effect upon binding, with Hill slopes being calculated at 3.5 and 2.6 for RAP2_2-15_ and HRE1_2-15_, respectively (Table 1). The interaction of AtPCO5.Ni(II) with ZPR2_2-15_ was determined to have a K_D_ of 692 ± 513 µM (Fig. 4f and Table 1)., similar to that seen for AtPCO4.Ni(II) with this substrate, but a cooperative effect was not seen for this interaction (Fig. 4e and Table 1). Interestingly, the interaction of AtPCO5.Ni(II) with VRN2_2-15_ (K_D_ 107 ± 30 µM, Fig. 4h) was determined to be much stronger than the equivalent interaction of AtPCO4.Ni(II) (K_D_ 495 ± 98 µM, Fig. 4g and Table 1) while retaining non-cooperative binding characteristics. This was unexpected given that VRN2 contains positively charged Arg residues at positions 3 and 7, which may have been expected to interact more favourably with the AtPCO4 hairpin loop than the AtPCO5 hairpin loop. This result suggests a complex range of interactions may be important in substrate binding to PCO enzymes. Indeed, when we investigated the interaction between AtPCO4.Ni(II) and a shorter peptidic form of the RAP peptide (RAP_2-7_) we found this to show a significantly weaker interaction than the RAP2_2-15_ peptide (Supplementary Fig. S4), demonstrating that interactions between the enzyme and peptide residues 8–15 play an important role in promoting substrate binding.

Finally, we wanted to further streamline our assay to maximise its utility and throughput by reducing the quantity of enzyme used in assays and the number of steps necessary for binding constant determination. We therefore repeated select assays with 2 µM AtPCO4 (maintaining AtPCO4.Ni(II) at 1:2 with 4 µM Ni(II)). Resulting binding curves (Supplementary Fig. S5) for RAP2_2-15_ and ZPR2_2-15_ were again found to fit models incorporating a Hill coefficient while VRN2_2-15_ was fit to a simple binding curve. Determined K_D_ values were found to be similar to those described above (Table 1), for example RAP2_2-15_ binding to AtPCO4.Ni(II) at 8 µM = 238.4 ± 11.6 µM and at 2 µM = 259.3 ± 23.3 µM. Interestingly, the cooperativity model fit for ZPR2_2-15_ no longer indicated cooperative binding when using 2 µM AtPCO4, with a Hill coefficient of 0.9 compared to 8 µM AtPCO4 with a Hill coefficient of 2 (Fig. 4e and Table 1). When the ZPR2_2-15_ fluorescence quenching data was fitted to a simple binding curve, the estimate K_D_ was lower (425.5 ± 70.0 µM). This may reflect a weaker propensity for ZPR2_2-15_ to promote cooperative binding than ERFVII substrates.

## Discussion

We have designed and optimised a plate-based assay that exploits the intrinsic fluorescence of Trp residues in AtPCO enzymes and the quenching of this fluorescence upon substrate binding to generate meaningful data on the affinity of PCOs for peptides representing biological substrates. In AtPCO4, this intrinsic fluorescence arises from Trp residues 121 and (predominantly) 217 which are not close to the presumed substrate binding site, implying that changes in AtPCO conformation may occur upon substrate binding. To prevent compounding impacts on Trp fluorescence of O_2_ (a cosubstrate of the PCOs) interacting with the enzymes, assays were set up under anaerobic conditions. The Trp fluorescence quenching effect was shown to be specific to peptides representing biological substrates. No meaningful inner filter effect was observed upon substrate addition.

We used the assay to investigate the binding to AtPCOs 4 and 5 of known substrates RAP2_2–15_, HRE1_2-15_, VRN2_2-15_ and ZPR2_2-15_. Substrates were 14-mer peptides, as used for previously reported biochemical investigations of AtPCO function, and therefore determined binding constants may not represent binding of full-length proteins in vivo. Nevertheless, the assays do allow comparison between substrates, which revealed some interesting observations. Overall, interactions with peptides representing VRN2_2-15_ and ZPR2_2-15_ were weaker than those for ERF-VII substrates, with the exception of AtPCO5 interaction with VRN2_2-15_. This is consistent with previously reported enzyme activity assays for AtPCO4 and may suggest that, in general, AtPCO interactions with ERFVIIs are more favourable than with non-ERFVII substrates^30^. Biologically, this could reflect the need for PCOs to readily bind to and oxidise ERFVII substrates under normoxic conditions, minimising expression of genes which promote anaerobic metabolism and thereby directing metabolism towards the more efficient oxidative phosphorylation. In addition, we noted an interesting cooperative effect on ligand binding to both AtPCO4 and AtPCO5, most notably upon binding ERFVII substrates. This was not observed when a shorter peptide, RAP2_2-7_, was used and may hint that the interactions between distal parts of the peptide ligand and enzyme interact to promote conformational change that confers a greater strength of interaction between enzyme and substrate at the active site; such ligand-driven conformational change has been observed in a range of metabolically important enzymes^37^.

Given the lack of structural information regarding substrate binding to AtPCO enzymes to date, this assay can be exploited to identify regions of the enzymes and substrates that are important in these interactions. PCOs are targets for manipulation as a strategy to alleviate the stressful impacts of acute hypoxia experienced by plants upon flooding^38^. Manipulation strategies may include mutation of substrate-binding residues and/or chemical inhibition of the enzymes; development of both strategies requires in vitro characterisation prior to implementation in vivo. This assay will prove a useful tool for quantifying the effects of PCO manipulation on the strength and selectivity of binding to substrates or substrate analogues.

## Electronic Supplementary Material

Below is the link to the electronic supplementary material.

Supplementary Material 1

Supplementary Material 2

## Data Availability

Data generated in this study are available within the article, Supplementary Information, and Source Data.
