# Supplementary material for: Measuring plant cysteine oxidase interactions with substrates using intrinsic tryptophan fluorescence
Source: Sci Rep. 2024 Dec 30;14:31960. doi: 10.1038/s41598-024-83508-y (PMC11685595; doi:10.1038/s41598-024-83508-y)
Supplement: Supplementary file 1 — Supplementary Material 1 [file 41598_2024_83508_MOESM1_ESM.docx]

**Measuring Plant Cysteine Oxidase Interactions with Substrates Using Intrinsic Tryptophan Fluorescence**

**Supplementary Information**

Dona M Gunawardana^a^, Daisy A. Southern^a^, Emily Flashman^b*^

^a^ – Department of Chemistry, University of Oxford, Oxford OX1 3TA, U.K.; dona.gunawardana@chem.ox.ac.uk, daisysouthern2000@gmail.com

^b^ – Department of Biology, University of Oxford, Oxford OX1 3RB, UK; emily.flashman@biology.ox.ac.uk

*Corresponding author

**Table of Contents:**

Figure S1 - Alignment of AtPCO sequences showing conserved Trp residues.

Figure S2 - AtPCO4.Ni(II) is inactive.

Figure S3 - Trp fluorescence quenching is due to binding of substrate.

Figure S4 - Binding of shorter version of RAP2_2-7_ shows weaker binding to AtPCO4.

Figure S5 - K_D_ determination of AtPCO4 with RAP2_2-15_, ZPR2_2-15_ and VRN2_2-15_.

Table S1- N-terminal sequences of known PCO substrates

**Supplementary Figures**


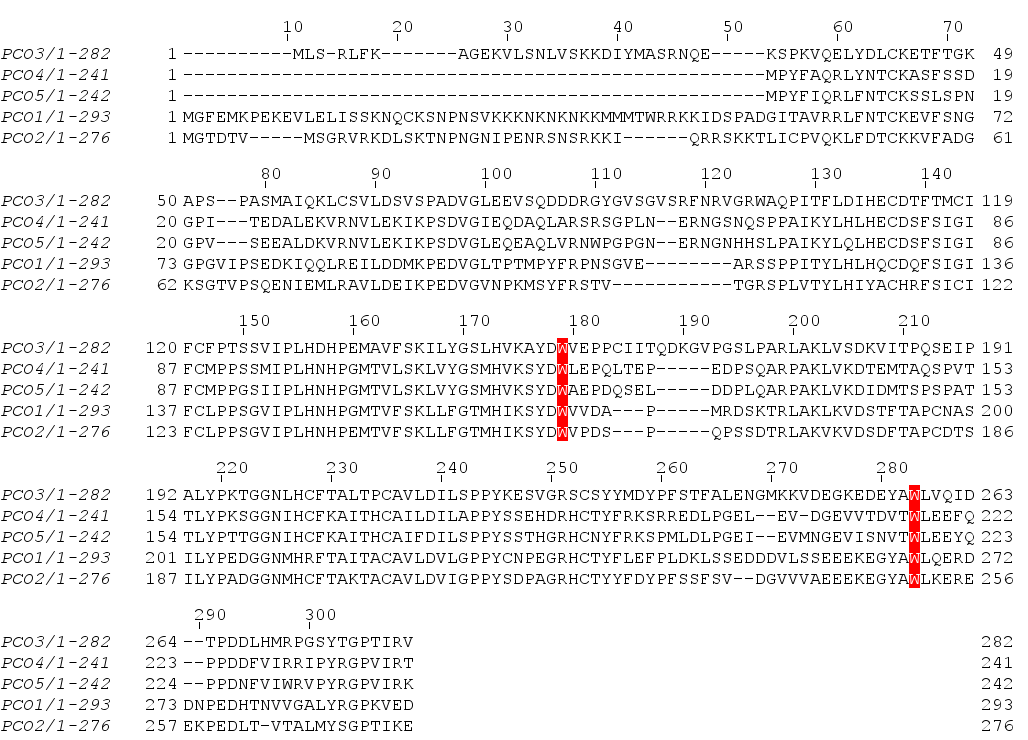


**Supplementary Figure S1. Alignment of AtPCO sequences showing conserved Trp residues.** Two conserved Trp residues 121 and 217 (AtPCO4 numbering) highlighted in red.


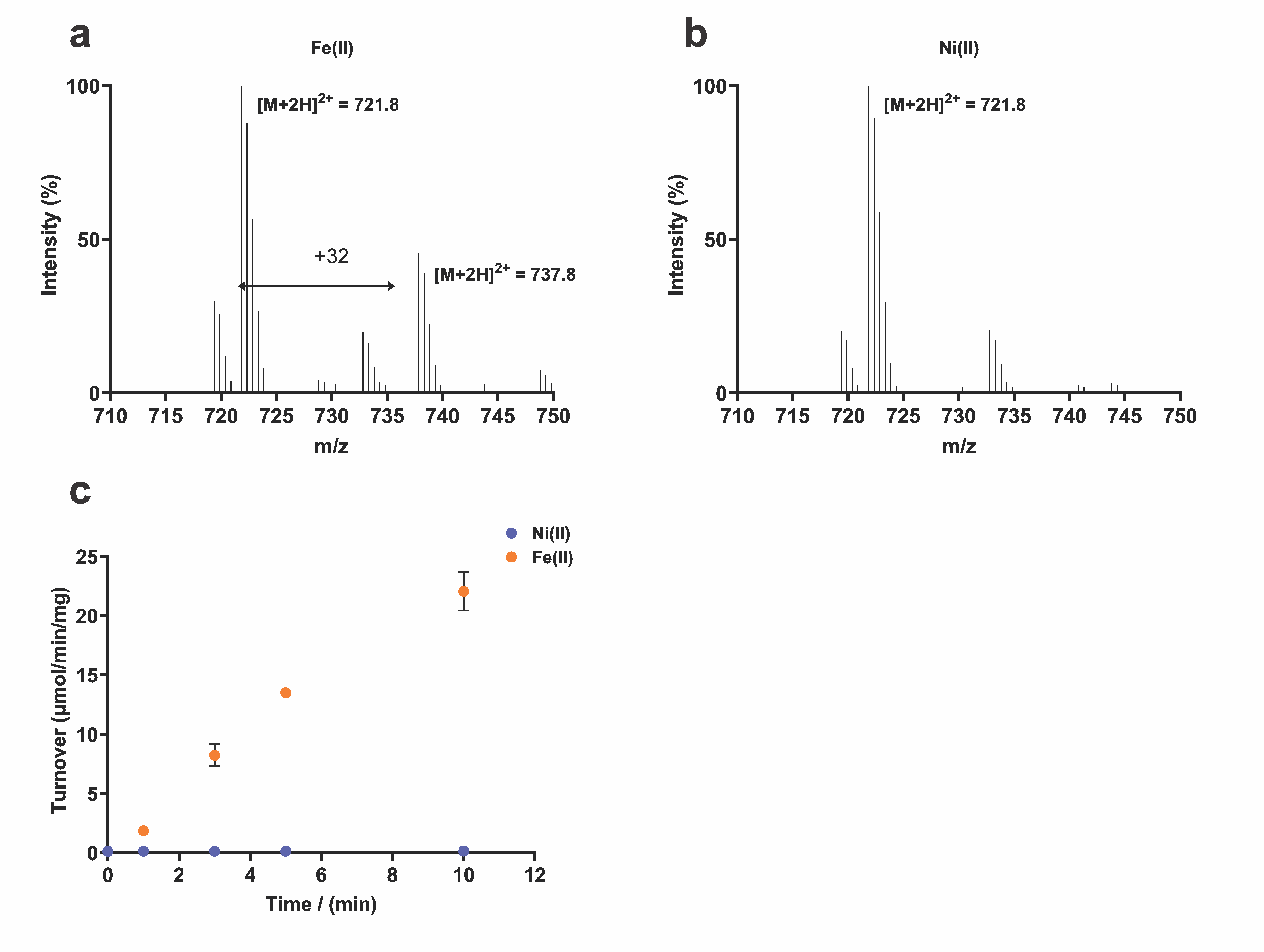


**Supplementary Figure S2. AtPCO4.Ni(II) is inactive.** Recombinant AtPCO4.Fe(II) catalyses oxidation of RAP2_2-15_, as demonstrated by the presence of a peak in the mass spectrum corresponding to a 32 Da mass increase on the RAP2_2-15_ ion (**a**). In contrast, AtPCO4.Ni(II) is inactive, as demonstrated by the absence of 32 Da mass increase on the RAP2_2-15_ ion (**b**) after 10 minutes (**c**).


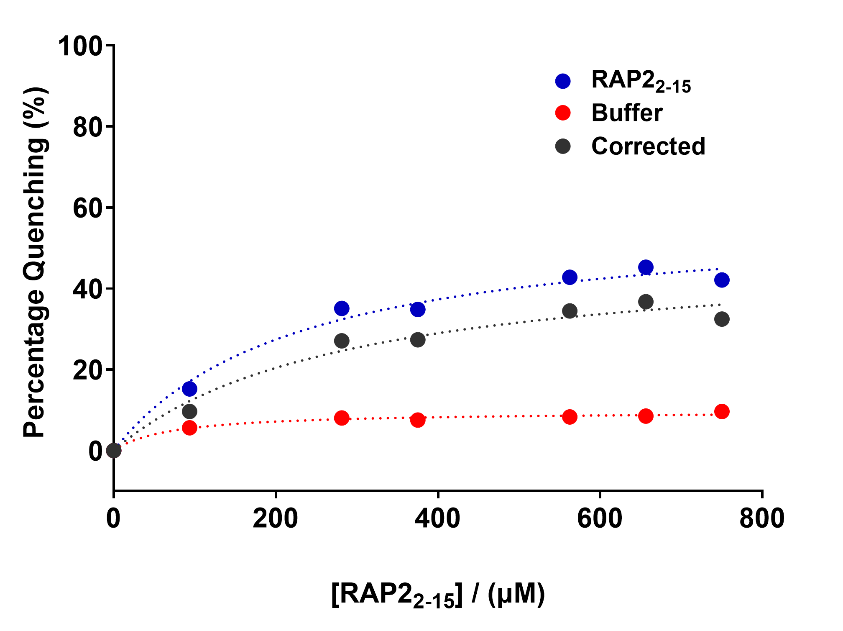


**Supplementary Figure S3. Trp fluorescence quenching is due to binding of substrate.** Trp fluorescence quenching as a % maximum fluorescence observed with 8 µM AtPCO4.Ni(II) upon addition of 3 µL increments of 46.8 µM RAP2_2-15_ solution or equivalent volumes of buffer control. A concentration-dependent effect confirms that the degree of Trp fluorescence quenching correlates with substrate binding.


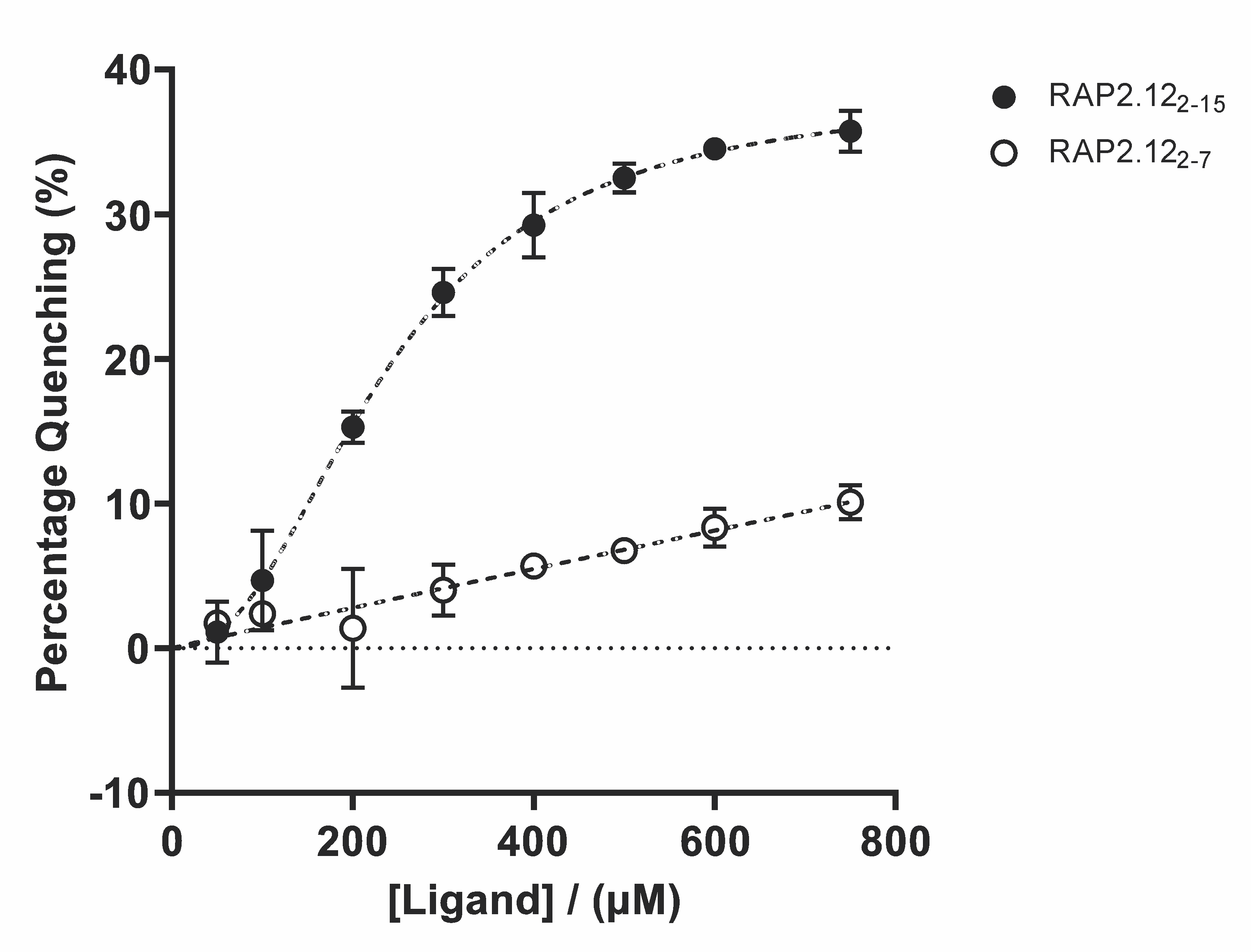


**Supplementary Figure S4. Binding of RAP2_2-7_ shows weaker binding to AtPCO4 than RAP2_2-15_.** Trp fluorescence quenching as a % maximum fluorescence observed with 8 µM AtPCO4.Ni(II) upon addition of 0-750 µM RAP2_2-7_ or 0-750 µM RAP2_2-15_. A concentration-dependent effect confirms that the degree of Trp fluorescence quenching with RAP2_2-7_ correlates with substrate binding, but the effect is much weaker than that observed for RAP2_2-15_. *Error bars display S.E (n=3).*


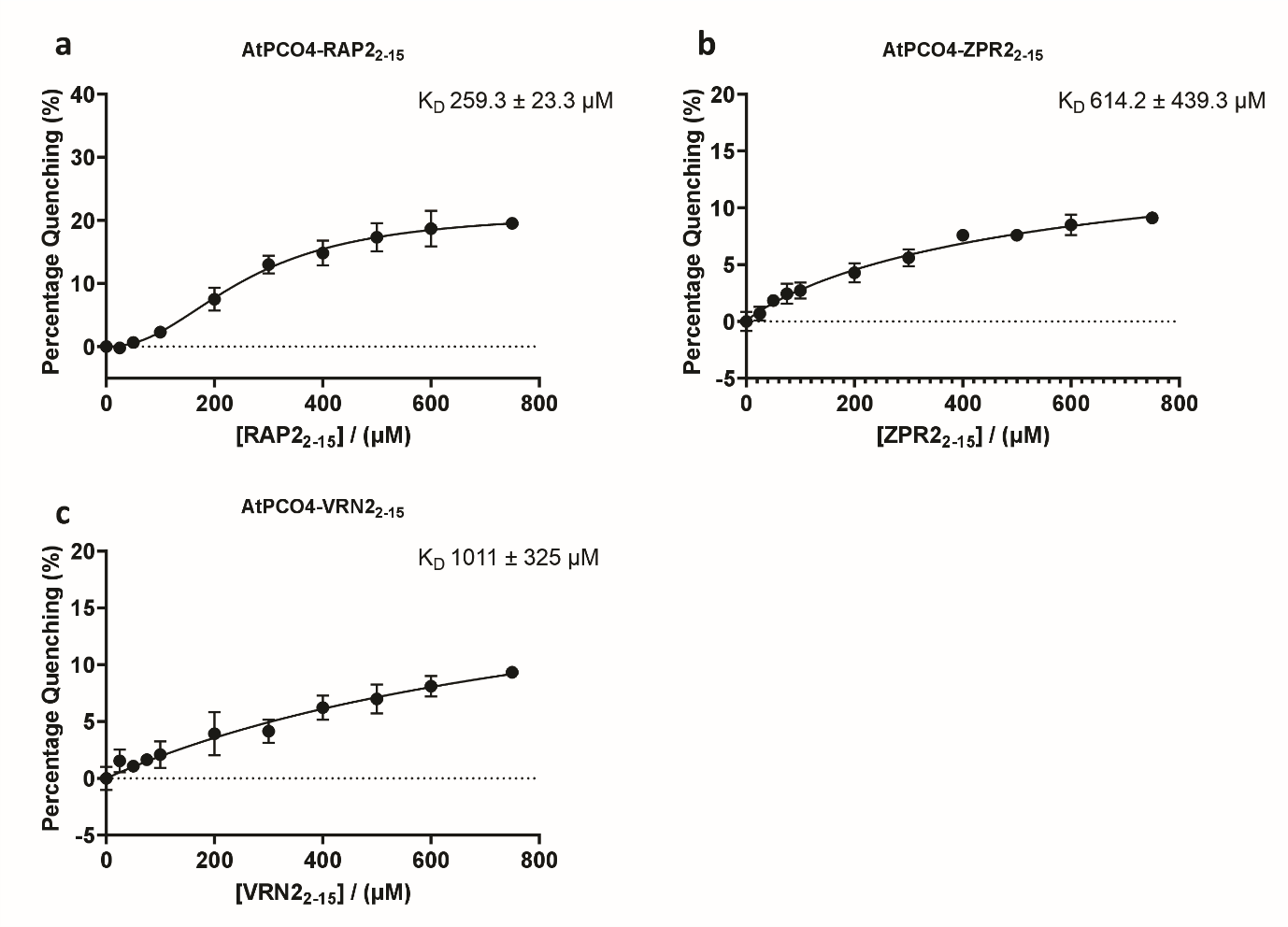


**Supplementary Figure S5. K_D_ determination of AtPCO4 with RAP2.12, ZPR2 and VRN2.** Trp fluorescence quenching of AtCO4.Ni(II) at 2 µM (with 4 µM Ni(II)) yield similar binding parameters to AtPCO4.Ni(II) at 8 µM (with 16 µM Ni(II)). For ZPR2 one site-specific binding with Hill slope (Y=Bmax*X^h/(Kd^h + X^h) model was fitted to be consistent with curve fitting at 8 µM AtPCO4.Ni(II). *Error bars display S.E (n=3).*

**Supplementary Tables**

| **Name** | **Gene locus** | **N-terminal sequence (initiating Met is removed)** |
| --- | --- | --- |
| RAP2.12 | *At1g53910* | CGGAIISDFIPPPR |
| RAP2.2 | *At3g14230* | CGGAIISDFIPPPR |
| RAP2.3 | *At3g16770* | CGGAIISDYIAPLV |
| HRE1 | *At1g72360* | CGGAILSDIIAPSA |
| HRE2 | *At2g47520* | CGGAIISDFIWSKS |
| VRN2 | *At4g16845* | CRQNCRAKSSPEEV |
| ZPR2 | *At3g60890* | CLTTSEPPFPDTDT |

**Supplementary Table 1. N-terminal sequences of known PCO substrates.** Residues 2-15 (Met_init_ removed) of *Arabidopsis thaliana* ERF-VIIs (RAP2.12, RAP2.2, RAP2.3, HRE1, HRE2), VRN2 and ZPR2 are shown. These sequences were used in peptidic form for binding assays.
